# Do we really need a surgery for hip fractures in elderly patients? Mortality rate and influencing factors

**DOI:** 10.1186/s42836-019-0009-1

**Published:** 2019-10-01

**Authors:** Kyu-Tae Hwang, Jun-Ki Moon, Young-Ho Kim

**Affiliations:** 1 Department of Orthopedic Surgery, Hanyang University Hospital, 222 Wangsimni-ro, Seongdong-gu, Seoul, 133-791 South Korea; 2 Department of Orthopedic Surgery, Hanyang University Guri Hospital, Gyomoon-dong 249-1, Guri city, Gyunggi-do 471-701 South Korea

**Keywords:** Elderly, Hip fracture, Mortality rate, Influencing factor

## Abstract

**Background:**

Hip fractures are associated with notable mortality rates in elderly patients. The purpose of the study was to evaluate the mortality rate and influencing factors associated with mortality in the elderly patients with hip fractures.

**Methods:**

Between October 2000 and December 2009, 807 elderly patients with hip fractures were enrolled in this study. There were 197 men and 610 women. The mean age at injuries were 78 years (range, 65–99 years). The fractures consisted of 390 femoral neck fractures and 417 intertrochanteric fractures. The mortality rate was evaluated between patients who underwent surgical and nonsurgical treatments. The influencing factors associated with mortality rate were evaluated statistically.

**Results:**

Overall, 691 (85.6%) patients treated surgically and 116 (14.4%) patients treated nonsurgically were included. The overall mortality rates one and two years after injuries were 16.6 and 39.4%, respectively. In surgical treatment group, the mortality rate one and two years after injuries were 12.0 and 35.7%, respectively. In nonsurgical treatment group, the mortality rates were 44.0 and 61.2%, respectively (*p* < 0.05). No significant difference was noted between the types of fractures and the time from injury to surgery. Regardless of surgical methods, a significantly higher mortality rate was observed in patients with heart disease, chronic renal disease, dementia, and cancer, or in patients with 3 or more comorbidities.

**Conclusions:**

In elderly patients with hip fractures, surgical treatments can decrease the mortality rate as compared with nonsurgical treatments. In addition, patients who had three or more comorbidities (heart disease, chronic renal failure, dementia, and history of cancer) are associated with a higher risk of mortality.

**Trial registration:**

Retrospectively registered.

## Introduction

The incidence of hip fractures in elderly patients is increasing as population ages [1]. Hip fractures are associated with notable morbidity and mortality in elderly patients. A recent epidemiological study showed that the proportion of patients with severe comorbidity increased from 10 to 19% between 1980 and 2014 [2]. In a 5-year period from 2010 through 2014, the standardized 30-day mortality rate and 31 to 365-day mortality rate were 10.55 and 19.28%, respectively. There are many factors that influence the mortality rate after hip fractures. Paksima et al. [3] stated that the mortality rate after hip fractures is related to patient’s age, ASA (American Society of Anesthesiologists) grades, postoperative complications, cancer history, chronic obstructive pulmonary disease, congestive heart failure, pre-injury ambulatory ability, and so on.

After hip fractures, most patients undergo surgery to reduce pain, to facilitate earlier ambulation, and to minimize complications, but some patients have nonsurgical treatments because of their medical condition and comorbidities [4]. Many studies have reported that the mortality rate after hip fracture was associated with various factors, but most studies were based on patients undergoing surgical treatment. There is little information regarding patients who received nonsurgical treatment.

In this retrospective study, we included all hip fractures regardless of treatments to evaluate the mortality rate and the influencing factors that affect mortality rates of the elderly patients one and 2 years after injury.

## Materials and methods

Among patients over the age of 65 who visited our institute for hip fracture between October 2000 and December 2009, 807 patients were evaluated after exclusion of multiple trauma and previous history of hip disease. There were 197 male (24.4%) and 610 (75.6%) female patients. The mean age at the time of injuries was 78 years (range, 65–99 years). The hip fractures consisted of 390 femoral neck fractures and 417 intertrochanteric fractures. The Statistics Korea (a governmental project of the Republic of Korea) was used to obtain precise data of mortality. Other medical data, in addition to survival and death rates, were analyzed retrospectively.

The patients were divided into a surgical group and a nonsurgical group to investigate the mortality rates one and 2 years after fractures. The patients of surgical group were treated with open reduction and internal fixation or hemi-arthroplasty. The patients who refused the recommended surgeries were allocated in nonsurgical group and treated with bed rest using an abduction brace. The age, sex, surgery, types of fracture, surgical method, time from injury to surgery, ASA grades, smoking history, types and number of comorbidities, and types of medical insurance in the patients were collected to assess the mortality-influencing factors.

Based on patient age, the patients were divided into a group of patients younger than 75 years of age, a group of patients aged between 75 and 85 years, and a group of patients older than 85 years of age. Based on the type of fractures, the patients were divided into femoral neck fracture group and intertrochanteric fracture group. Based on the surgical techniques, the patients were divided into hemi-arthroplasty group and internal fixation group. Based on the time interval from injury to surgery, the patients were divided into a group within 5 days and a group after 5 days. Based on ASA grades, patients were divided into a lower-grade group (I, II) and a higher-grade group 66 (III, IV). The underlying diseases such as congestive heart failure, ischemic heart disease, dementia, chronic kidney disease, hypertension, diabetes, chronic obstructive pulmonary disease, cancer, liver cirrhosis, rheumatoid arthritis, and Parkinson’s disease were also allocated. Based on the number of comorbidities, the patients were divided into 0–2 comorbidities group and 3 and above comorbidities group. Based on patient’s medical reimbursement coverages, the patients were divided into medical insurance group and national medical care group. The national medical care refers to the policy issued by the Korean government for protection of patients with low incomes, which provides basic treatments, surgeries, medicines, and hospital transfer for free. Medical insurance is a co-pay system priced at a percentage of patient’s salary. It covers every patient residing in Korea by paying a portion of medical cost.

SPSS 16.0 statistical software (IBM Corp, Armonk, NY USA) was used for analysis. Kaplan-Meier survival analysis was used for comparison and analysis of the mortality rates after fractures in both surgical and nonsurgical groups, and a chi-squared test and multivariate logistic regression were performed. A *p* value < 0.05 was considered to show statistical significance.

## Results

There were 691 patients (85.6%) who underwent surgical treatments, and the remaining 116 patients (14.4%) had nonsurgical treatments including some patients who refused surgical treatments due to economic or insurance issues. The mortality rates of all patients one and 2 years after injury were 16.6 and 39.4%, respectively. Of the 691 patients who did undergo surgical treatment, the mortality rates one and 2 years after injury were 12.0 and 35.7%, respectively. Of the 116 patients with non-surgical treatments, the mortality rates one and 2 years after injury were 44.0 and 61.2%, respectively. Thus, the surgical group had a significantly lower mortality rate (*p* = 0.001) (Table 1).

**Table 1:** Mortality rates of surgical and nonsurgical treatments

	Total	Surgical treatment	Nonsurgical treatment	*p* value
Patient number	807	691	116	
Death 1 year after the traumatic event	134 (16.6%)	83 (12.0%)	51 (44.4%)	< 0.001
Death 2 years after the traumatic event	318 (39.4%)	247 (35.7%)	71 (61.2%)	< 0.001

**Fig. 1:**
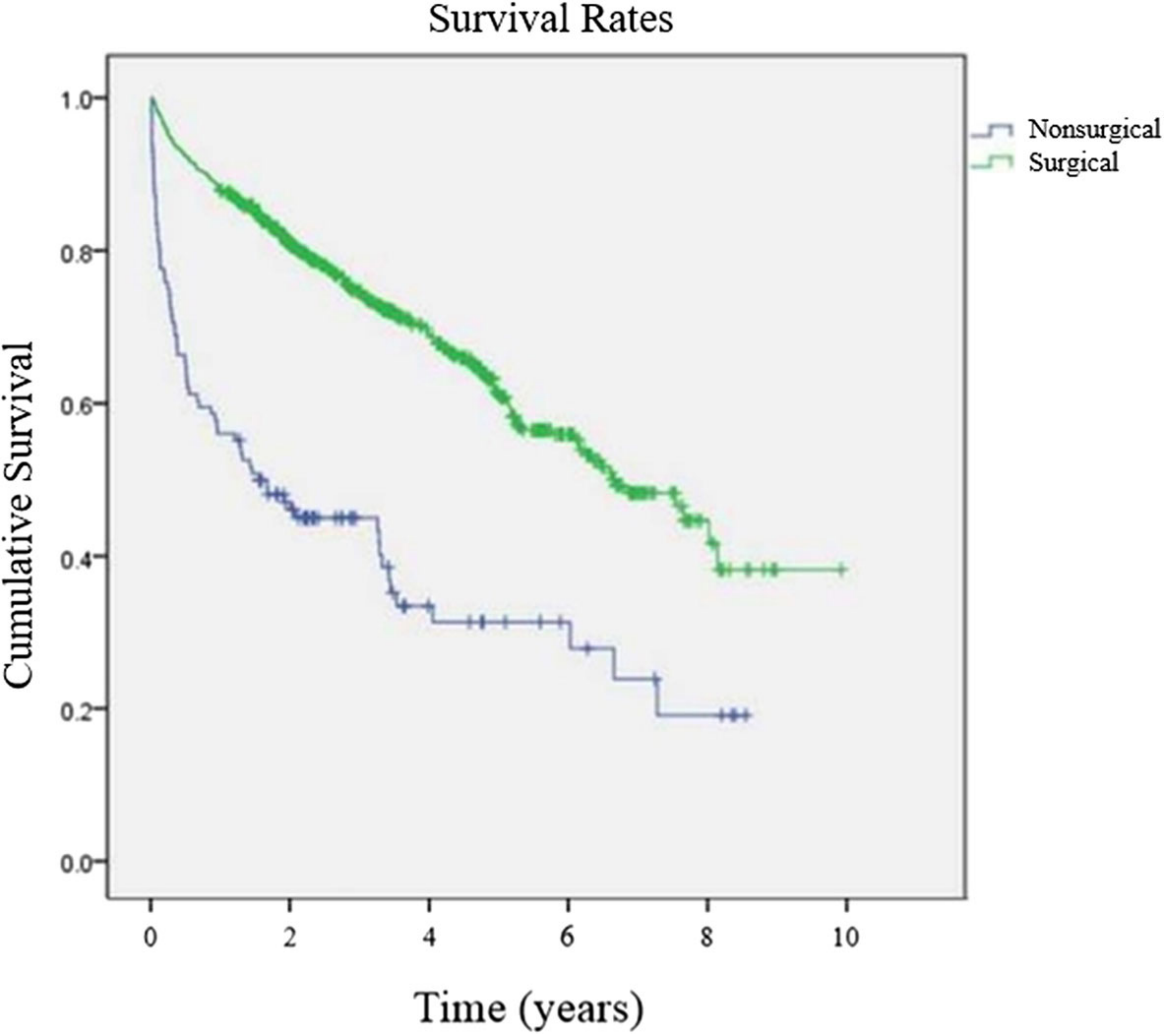
Kaplan-Meier survival rates between surgical and nonsurgical treatment groups

The various factors that may affect mortality rates are shown in Table 2. One and 2 years after injury, the mortality was significantly higher in male patients (*p* < 0.001, *p* = 0.001). The mortality rates were significantly higher one and 2 years after injury in the older aged group (*p* < 0.001). The mortality rates at one and 2 years after injury were higher in the nonsurgical group (*p* < 0.001). The Kaplan-Meier survival analysis showed that the 5-year survival rate of the surgical group was 60.8% and that of the nonsurgical group was 31.3%. Thus, the surgical group showed a higher survival rate (Fig. 1). The mortality rate at 1 year after injury of the lower ASA grade group was lower than that of higher ASA grade group (*p* < 0.001).

**Table 2:** Influencing factors associated with mortality 1 and 2 years after the traumatic event

Factors	Death 1 year after traumatic event	*p*-value	Death 2 years after traumatic event	*p*-value
Sex		< 0.001		0.001
Female	85 (13.9%)		221 (36.2%)	
Male	49 (24.9%)		97 (48.2%)	
Age		< 0.001		< 0.001
65–75	31 (10.2%)		94 (31%)	
75–85	77 (19.2%)		148 (36.9%)	
> 85	26 (25.2%)		56 (54.4%)	
Treatments		< 0.001		< 0.001
Surgical	83 (12%)		247 (35.8%)	
Nonsurgical	51 (44%)		71 (61.2%)	
ASA grade		< 0.001		0.081
I, II	21 (6.7%)		98 (31.1%)	
III, IV	62 (16.5%)		149 (39.6%)	
Fracture region of femur		0.886		0.268
Neck	64 (16.4%)		146 (37.4%)	
Intertrochanteric	70 (16.8%)		172 (41.3%)	
Time to surgery		0.764		0.615
< 5 days	19 (15.8%)		46 (38.3%)	
≥ 5 days	96 (16.8%)		231 (40.5%)	
No. of comorbidity		< 0.001		0.056
< 3	104 (14.8%)		269 (38.2%)	
≥ 3	30 (29.4%)		49 (48.0%)	
Smoking History		< 0.001		0.030
Smoking	41 (28.08%)		69 (47.26%)	
Non-smoking	92 (13.94%)		248 (37.58%)	
Insurance		0.004		0.134
Medical insurance	114 (15.45%)		285 (38.62%)	
NMC	20 (28.99%)		33 (47.83%)	

*NMC* national medical care

Data concerning fracture types and treatment options are shown in Table 3. According to the surgical methods, a statistically significant difference in mortality rates was observed for intertrochanteric fractures 1 year after fracture, but no significant difference in the mortality rates was observed between the two groups 2 years after fracture (*p* = 0.012, *p* = 0.519). In patients with femoral neck fracture, according to the surgical method, mortality rates at both one and 2 years after injury were not significantly different (*p* = 0.574, *p* = 0.856).

**Table 3:** Mortality rates of surgical techniques 1 and 2 years after the traumatic event

Variables	Death 1 year after the traumatic event	*p*-value	Death 2 years after the traumatic event	*p*-value
Neck fractures		0.574		0.856
Arthroplasty	41 (13.2%)		105 (33.9%)	
Internal fixation	3 (9.7%)		10 (32.3%)	
Intertrochanteric fractures		0.012		0.519
Arthroplasty	22 (16.54%)		53 (39.85%)	
Internal fixation	17 (7.83%)		79 (36.41%)	
Overall		0.017		0.954
Arthroplasty	63 (14.2%)		158 (35.7%)	
Internal fixation	20 (8.1%)		89 (35.9%)	

The mortality rates of medical comorbidities are shown in Table 4. The mortality rate 1 year after injury was significantly higher in patients with ≥ 3 comorbidities than patients with less than three comorbidities (*p* = 0.001). In patients with associated comorbidities such as congestive heart failure, ischemic heart disease, dementia, chronic kidney disease, and cancer, the mortality rates one and 2 years after injury were significantly higher.

**Table 4:** Mortality rates of medical comorbidities

Medical comorbidity	Total	Death 1 year after the traumatic event	*p*-value	Death 2 years after the traumatic event	*p*-value
CHF	42	16 (38.1%)	< 0.001	24 (57.1%)	0.016
IHD	61	17 (27.0%)	0.021	26 (41.3%)	0.752
Dementia	81	23 (28.4%)	0.003	49 (60.5%)	< 0.001
CKD	31	11 (35.5%)	0.004	17 (54.8%)	0.073
DM	239	38 (15.9%)	0.727	87 (36.4%)	0.257
Hypertension	483	75 (15.5%)	0.316	189 (39.1%)	0.845
COPD	95	21 (22.1%)	0.125	41 (43.2%)	0.426
Cancer	38	20 (52.6%)	< 0.001	26 (69.4%)	< 0.001
LC	12	3 (25.0%)	0.431	6 (50.0%)	0.449
RA	22	2 (9.1%)	0.337	6 (27.3%)	0.238
Parkinson’s disease	11	1 (9.1%)	0.507	4 (36.3%)	0.842

*CHF* congestive heart failure, *IHD* ischemic heart disease, *CKD* chronic kidney disease, *DM* diabetes mellitus, *COPD* chronic obstructive pulmonary disease, *LC* liver cirrhosis, *RA* rheumatoid arthritis

The predictors of mortalities 1 and 2 years after injury are shown in Table 5. The mortality rate 1 year after injury was related to age, surgical treatment, ASA grades, the number of associated comorbidities, and smoking. By contrast, the mortality rate 2 years after injury was related to sex, age, and surgical treatment. Among the various factors, surgical treatment had the greatest effect on the mortality rate one and 2 years after injury (Odds ratio: 4.39 and Odds ratio: 2.52, respectively).

**Table 5:** Predictors of mortalities 1 and 2 years after the traumatic events

Factors	Death 1 year after the traumatic event			Death 2 years after the traumatic event		
	OR	95% CI	*p*-value	OR	95% CI	*p*-value
Male	1.40	0.82–2.39	0.217	1.60	1.06–2.40	0.024
Age ≥ 75	2.00	1.22–3.28	0.006	1.52	1.09–2.13	0.014
Surgical treatment	4.39	2.61–7.35	0.000	2.52	1.56–4.08	0.000
ASA grade III, IV	2.25	1.33–3.81	0.003	1.35	0.96–1.89	0.086
Neck fracture	1.12	0.72–1.74	0.608	0.93	0.67–1.28	0.637
No. of comorbidity ≥ 3	1.90	1.07–3.39	0.030	1.39	0.85–2.28	0.184
Smoking history	2.00	1.14–3.53	0.016	1.19	0.76–1.87	0.454
Medical aid	1.16	0.57–2.37	0.683	0.89	0.49–1.63	0.710
Time to surgery ≥ 5 days	1.00	0.56–1.78	0.998	1.08	0.71–1.64	0.708

*OR* odds ratio, *CI* confidence interval, *ASA* American Society of Anesthesiologists

## Discussion

With the aging society, the elderly population is increasing, and the hip fractures in the elderly are gradually increasing. In elderly patients with osteoporosis, hip fractures are frequently the results of low-energy traumas such as falling on the ground. Hip fracture in the elderly is an important problem that can lead to death. Many studies reported the mortality rates of hip surgery after 1 year ranged from 12.7 to 29.2% [5–8]. In our study, the total mortality rate 1 year after injury was 16.6%, similar to those reported previously. The mortality rates 1 year after surgery was 12.0% in surgical group and 44.0% in nonsurgical group with significant difference. The overall mortality rate 2 years after injury was 39.4%, and the mortality rates 2 years after injury were significantly different between surgical group and nonsurgical group. The mortality rates 1 year after injury was similar to that of the normal population [9], but the mortality rate 2 years after injury was higher than that 1 year after injury. We thought that these results were associated with various factors in elderly patients, such as age, sex, type of fractures, comorbidities, and surgical techniques.

Among the various factors related to mortality rate, age has been reported as one of the most influencing factors after hip fracture. Paksima et al. [3] and Miller [10] reported that the mortality rate increased with increasing age, but White et al. [9] and Cornwall et al. [11] found that the mortality rate and age showed an inverse-relation (that the mortality rate does not differ with age). Our study showed higher mortality rates both one and 2 years after injury in patient ≥75 years of age.

Based on gender, Kenzora et al. [12] reported no difference in the mortality rates between male and female patients. However, Miller [10] reported a higher mortality rate in male patients. In our multiple regression analysis, the mortality rate increased 2 years after injury only in male patients. However, this result may be associated with other factors such as the difference in average life expectancy of male and female patients. Therefore, mutiple factors should be analysed.

In general, intertrochanteric fracture was caused more frequently by higher energy trauma as compared with femoral neck fracture. Therefore, intertrochanteric fracture induces more bleeding and requires a longer operation time, more complicated surgical methods and requires avoiding full weight-bearing exercise after the operation. As a result, patients with intertrochanteric fracture showed a higher mortality rate than patients with femoral neck fracture [13–15]. However, there were also reports that there was no difference in mortality rates between the two fracture types [12, 15]. In our study, there was no statistically significant difference in mortality rates according to type of fracture. In previous reports, Hossain et al. [16] reported that nonsurgical treatment can be an appropriate choice for patients who are not suitable for surgery after fracture, and no significant difference in mortality rates or functional results was observed between the surgical and nonsurgical groups. However, Yoon et al. [4] reported that the higher mortality rate in the nonsurgical group was caused by financial issues, and they showed a higher mortality rate and a serious loss of functions. In this study, significantly higher mortality rates were observed in the nonsurgical group one and 2 years after injury. In addition, results of multiple regression analysis indicated that surgery was the greatest factor affecting mortality rate.

According to surgical method employed, Garden [17] reported higher mortality rates for hemiarthroplasty than for internal fixation, however, Sikorski and Barrington [18] reported the opposite results. Several studies reported that there was no difference in mortality rates between surgical methods [17, 18]. Hemiarthroplasty takes more time during surgery than internal fixation, and produces more bleeding, which leads to a higher acute mortality rate, but an advantage is that the non-weight-bearing period for fracture healing can be shortened. This study found a significantly higher mortality rate 1 year after injury for the hemiarthroplasty group, but no significant difference was revealed 2 years after injury. The reason for the higher mortality rate 1 year after injury is probably that hemiarthroplasty was preferentially selected for patients of age ≥ 75, when there was evidence of poor bone quality, and comminuted fractures were present.

Generally, hemiarthroplasty may not be appropriate for patients younger than 70 years, because of possibility of excessive degeneration of acetabular articular cartilage. This degenerative change increased the rate of revision surgery to as high as 26% 5 years after surgery [19]. However, a recent big data research on femoral neck fractures conducted by Eskildsen et al. [20], showed that overall revision rates of hemiarthroplasty and total hip arthroplasty were similar in patients aged between 65 and 69 years. Blomfeldt et al. [21] reported that the bipolar hemiarthroplasty may be sufficient for elderly patients with lower functional demands, because the total hip arthroplasty results in increased blood loss and longer operating time. Therefore, we believe that hemiarthroplasty is a useful treatment option for the limited purposes in the frailest patients (ASA grade III/IV), even though the patients are under 70 years of age.

Although various factors affect mortality rate in hip fractures, many studies found the associated comorbidities were the key factor. Lehner et al. [22] reported that the mortality rate was higher in patients with two or more associated comorbidities. Kilci et al. [5] reported that the mortality rate increases with ASA grades and the number of comorbidities. Our study supported their findings on the baseline of 1 year after injury, but the mortality rate decreased to the level of normal population 2 years after injury. Other factors, like comorbidities and ASA grades may affect mortality rates. Regarding the results of mortality rate based on each comorbidity, several studies found that heart diseases are the greatest factor affecting mortality rate [23, 24]. In this study, patients with congestive heart failure, dementia, and cancer had higher mortality rates than those who did not have comorbidities. Patients with ischemic heart diseases and chronic kidney diseases had a higher mortality rate 1 year after injury, compared with patients who did not have such conditions. We did not find other comorbidities that affected the mortality rate.

Regarding the time from injury to operation, Zuckerman et al. [25] reported that the mortality rate 1 year after injury increased if the surgery was performed 3 days after injury. In this study, the patients were divided into two groups based on the five-day point after injury, we found the mortality rates one and 2 years after injury are similar in both groups. In this study, if the number of comorbidities was low (< 3), it was possible to perform the surgery within 5 days after injury, but more comorbidities are associated with more preoperative evaluations and examinations and prolonged time between injury and surgery. However, we were able to determine the exact status and mortality rates of the two groups.

Our study has some limitations. First, it is unable to determine the direct relations between hip fracture and the cause of death because many difficulties exist. Second, accurate comparative analysis was impossible because of the too small a number of patients in nonsurgical group. Third, multiple bias may exist based on a single-center observation. This is not a blinded study, and the patients were not randomly allocated.

## Conclusions

In elderly patients with hip fractures, surgical treatments can decrease the mortality rate compareing with nonsurgical treatments. In addition, patients who had three or more comorbidities (heart disease, chronic renal failure, dementia, and history of cancer) are associated with a higher risk of mortality.

## Data Availability

The datasets used and/or analysed during the current study are available from the corresponding author on reasonable request.

## Abbreviation

ASA: American Society of Anesthesiologists
